# 

**Published:** 1893-09-30

**Authors:** 


### CONTENTS.

**The Student at the Apex** - - - - - - - - - 417

**Around the Hospitals** - - - - - - - - - 418

**Great Achievements Under Suffering.**—IV. The Blind Observer - - - - - - - - - - - - 419

**The History and Capabilities of Herbal Simples.** By W. T. FERNIE, M.D.—The Gooseberry - - - - - - 420

**Famous Poisoners in Fiction.**—VI. A Mother's Love - - 421

**Gleanings in Science and Medicine** - - - - - 422

**Annotations.**—King's College and Public Health—Lord Meath Physical Educator—Curiosities of Cerebration - - - - 423

**Notes and News** - - - - - - - - - - 424

**Scraps and Gleanings** - - - - - - - - - 432

**The Book World of Medicine and Science** - - - - vii

**Medical Vacancies and Appointments** - - - - - x

**The Hospital Clinic—**

ROYAL ORTHOPÆDIC HOSPITAL.—Caries of the Spine - - - 425

INGROWING TOE-NAIL.—I. Pathology - - - - - - - 426

NEW DRUGS AND PREPARATIONS.—Subcutaneous Injections of Strychnine - - - - - - - - - - - - 427

NEW APPLIANCES AND THINGS MEDICAL.—Oleusaban Disinfectants—Whole Wheat Flour with Hypophosphites - - - 428

EDITOR'S LETTER-BOX.—How to Diminish the Numbers of Doctors 428

**The Institutional Workshop—**

HOSPITAL ADMINISTRATION.—V. Special Hospitals : Ophthalmic and Miscellaneous—Cost of Management - - - - 429

THE STORY OF THE INSANE FROM YEAR TO YEAR - - - - 429

PRACTICAL DEPARTMENTS.—Dressing Waggon - - - - 431

THE CARE OF THE FEEBLE-MINDED.—II. In Later Life - - 431

### EXTRA SUPPLEMENT.—THE NURSING MIRROR.

**News from the Nursing World.**—Pension and Sick Assurance — Our Christmas Competitions — Notes and Queries—Is it Worth Doing ?—Hospitality to Nurses—Mistaken Economy—Work in Scotland—Scientific Cooking—Obvious Dangers—Short Items - - - - - - - cclxi

**Insanity Among Coloured Races.**—African Asylums - cclxiii

**A Hint to Ward Nurses** - - - - - - - - cclxiii

**Nursing in Paris.**—II. Inside the Hotel Dieu - - - cclxiv

**Modern Nurses** - - - - - - - - - - - cclxv

**Where to Go** - - - - - - - - - - - - cclxv

**Nursing in South Africa** - - - - - - - - cclxv

**District Nursing** - - - - - - - - - - cclxvi

**Everybody's Opinion.**—Watford District Cottage Hospital cclxvi

**Notes from Philadelphia.**—Nursing at the Methodist Episcopal Hospital - - - - - - - - - - cclxvii

**For Reading to the Sick.**—The Cross - - - - cclxvii

**Minor Appointments** - - - - - - - - - cclxvii

**The Muses' Looking Glass.**—Pitfalls in Pamphlets - cclxviii

**Notes and Queries** - - - - - - - - - - cclxviii

**The Book World for Women and Nurses** - - - cclxix

